# Genome-Wide Association Study Confirming Association of HLA-DP with Protection against Chronic Hepatitis B and Viral Clearance in Japanese and Korean

**DOI:** 10.1371/journal.pone.0039175

**Published:** 2012-06-21

**Authors:** Nao Nishida, Hiromi Sawai, Kentaro Matsuura, Masaya Sugiyama, Sang Hoon Ahn, Jun Yong Park, Shuhei Hige, Jong-Hon Kang, Kazuyuki Suzuki, Masayuki Kurosaki, Yasuhiro Asahina, Satoshi Mochida, Masaaki Watanabe, Eiji Tanaka, Masao Honda, Shuichi Kaneko, Etsuro Orito, Yoshito Itoh, Eiji Mita, Akihiro Tamori, Yoshikazu Murawaki, Yoichi Hiasa, Isao Sakaida, Masaaki Korenaga, Keisuke Hino, Tatsuya Ide, Minae Kawashima, Yoriko Mawatari, Megumi Sageshima, Yuko Ogasawara, Asako Koike, Namiki Izumi, Kwang-Hyub Han, Yasuhito Tanaka, Katsushi Tokunaga, Masashi Mizokami

**Affiliations:** 1 Research Center for Hepatitis and Immunology, National Center for Global Health and Medicine, Ichikawa, Chiba, Japan; 2 Department of Human Genetics, The University of Tokyo, Bunkyo-ku, Tokyo, Japan; 3 Department of Virology and Liver Unit, Nagoya City University Graduate School of Medical Sciences, Nagoya, Aichi, Japan; 4 Department of Internal Medicine, Yonsei University College of Medicine, Seoul, South Korea; 5 Department of Internal Medicine, Hokkaido University Graduate School of Medicine, Sapporo, Japan; 6 Department of Internal Medicine, Teine Keijinkai Hospital, Sapporo, Japan; 7 Department of Gastroenterology and Hepatology, Iwate Medical University, Morioka, Japan; 8 Division of Gastroenterology and Hepatology, Musashino Red Cross Hospital, Tokyo, Japan; 9 Division of Gastroenterology and Hepatology, Saitama Medical University, Saitama, Japan; 10 Department of Gastroenterology, Kitasato University School of Medicine, Sagamihara, Kanagawa, Japan; 11 Department of Medicine, Shinshu University School of Medicine, Matsumoto, Japan; 12 Department of Gastroenterology, Kanazawa University Graduate School of Medicine, Kanazawa, Japan; 13 Department of Gastroenterology, Nagoya Daini Red Cross Hospital, Nagoya, Japan; 14 Molecular Gastroenterology and Hepatology, Kyoto Prefectural University of Medicine, Kyoto, Japan; 15 Department of Gastroenterology and Hepatology, National Hospital Organization Osaka National Hospital, Osaka, Japan; 16 Department of Hepatology, Osaka City University Graduate School of Medicine, Osaka, Japan; 17 Second Department of Internal Medicine, Faculty of Medicine, Tottori University, Yonago, Japan; 18 Department of Gastroenterology and Metabology, Ehime University Graduate School of Medicine, Ehime, Japan; 19 Gastroenterology and Hepatology, Yamaguchi University Graduate School of Medicine, Yamaguchi, Japan; 20 Division of Hepatology and Pancreatology, Kawasaki Medical College, Kurashiki, Japan; 21 Division of Gastroenterology, Department of Medicine, Kurume University School of Medicine, Fukuoka, Japan; 22 Central Research Laboratory, Hitachi Ltd., Kokubunji, Tokyo, Japan; Drexel University College of Medicine, United States of America

## Abstract

Hepatitis B virus (HBV) infection can lead to serious liver diseases, including liver cirrhosis (LC) and hepatocellular carcinoma (HCC); however, about 85–90% of infected individuals become inactive carriers with sustained biochemical remission and very low risk of LC or HCC. To identify host genetic factors contributing to HBV clearance, we conducted genome-wide association studies (GWAS) and replication analysis using samples from HBV carriers and spontaneously HBV-resolved Japanese and Korean individuals. Association analysis in the Japanese and Korean data identified the *HLA-DPA1* and *HLA-DPB1* genes with *P_meta_* = 1.89×10^−12^ for rs3077 and *P_meta_* = 9.69×10^−10^ for rs9277542. We also found that the *HLA-DPA1* and *HLA-DPB1* genes were significantly associated with protective effects against chronic hepatitis B (CHB) in Japanese, Korean and other Asian populations, including Chinese and Thai individuals (*P_meta_* = 4.40×10^−19^ for rs3077 and *P_meta_* = 1.28×10^−15^ for rs9277542). These results suggest that the associations between the *HLA-DP* locus and the protective effects against persistent HBV infection and with clearance of HBV were replicated widely in East Asian populations; however, there are no reports of GWAS in Caucasian or African populations. Based on the GWAS in this study, there were no significant SNPs associated with HCC development. To clarify the pathogenesis of CHB and the mechanisms of HBV clearance, further studies are necessary, including functional analyses of the HLA-DP molecule.

## Introduction

Overall, one-third of the world’s population (2.2 billion) is infected with hepatitis B virus (HBV), and about 15% of these are chronic carriers. About 75% of the chronic carriers live in the east-south Asia and east pacific area, and there are 1.3–1.5 million chronic carriers living in Japan [1]. Of chronic carriers, 10–15% develop liver cirrhosis (LC), liver failure and hepatocellular carcinoma (HCC), and the remaining individuals eventually achieve a state of nonreplicative infection, resulting in hepatitis B surface antigen (HBsAg) negative and hepatitis B core antibody (anti-HBc) positive, i.e. HBV-resolved individuals [2]–[3]. In Japan, although the major route of HBV transmission was perinatal transmission and horizontal transmission in early childhood, infant HBV carriers have successfully been reduced since 1986 through a selective vaccination policy by the Japanese government [4]–[7]. However, the prevalence of HBV genotype A in acute HBV (AHB) infection has increased markedly since 2000, reaching approximately 52% in 2008 due to the lack of a universal HB vaccination, and around 10% of AHB cases could be persistent infection [8]–[9]. Viral factors, as well as host factors, are thought to be associated with persistent HB infection.

In 2009, significant associations between chronic hepatitis B (CHB) and a region including *HLA-DPA1* and *HLA-DPB1* were identified using 786 Japanese individuals having CHB and 2,201 control individuals through a two-stage genome-wide association study (GWAS) [10]. The same group was also subjected to a second GWAS using a total of 2,667 Japanese persistent HBV infection cases and 6,496 controls, which confirmed significant associations between the *HLA-DP* locus and CHB, in addition to associations with another two SNPs located in the genetic region including the *HLA-DQ* gene [11]. The associations between *HLA-DP* variants with HBV infection were replicated in other Asian populations, including Thai and Han Chinese individuals [10], [12]–[13]. With regard to HBV clearance, the association between the human leukocyte antigen (HLA) class II allele and clearance of HBV was confirmed by the candidate gene approach in African, Caucasian and Asian populations [14]–[18]. However, in a previous GWAS using samples of Japanese CHB and control individuals, the clinical data on HBV exposure in the control individuals were unknown, and this may have led to bias. Moreover, there have been no reports of GWAS using samples from HBV carriers and HBV-resolved individuals to identify host genetic factors associated with HBV clearance other than HLA class II molecules.

Here, we performed a GWAS using samples from Japanese HBV carriers, healthy controls and spontaneously HBV-resolved individuals in order to confirm or identify the host genetic factors related to CHB and viral clearance. In the subsequent replication analysis, we validated the associated SNPs in the GWAS using two independent sets of Japanese and Korean individuals. In our study, healthy controls were randomly selected with clinically no evidence of HBV exposure, therefore, HBV-resolved individuals were prepared to clearly identify the host genetic factors related with CHB or HBV clearance.

## Results

### Protective Effects Against Chronic Hepatitis B in Japanese and Korean Individuals

**Figure 1:**
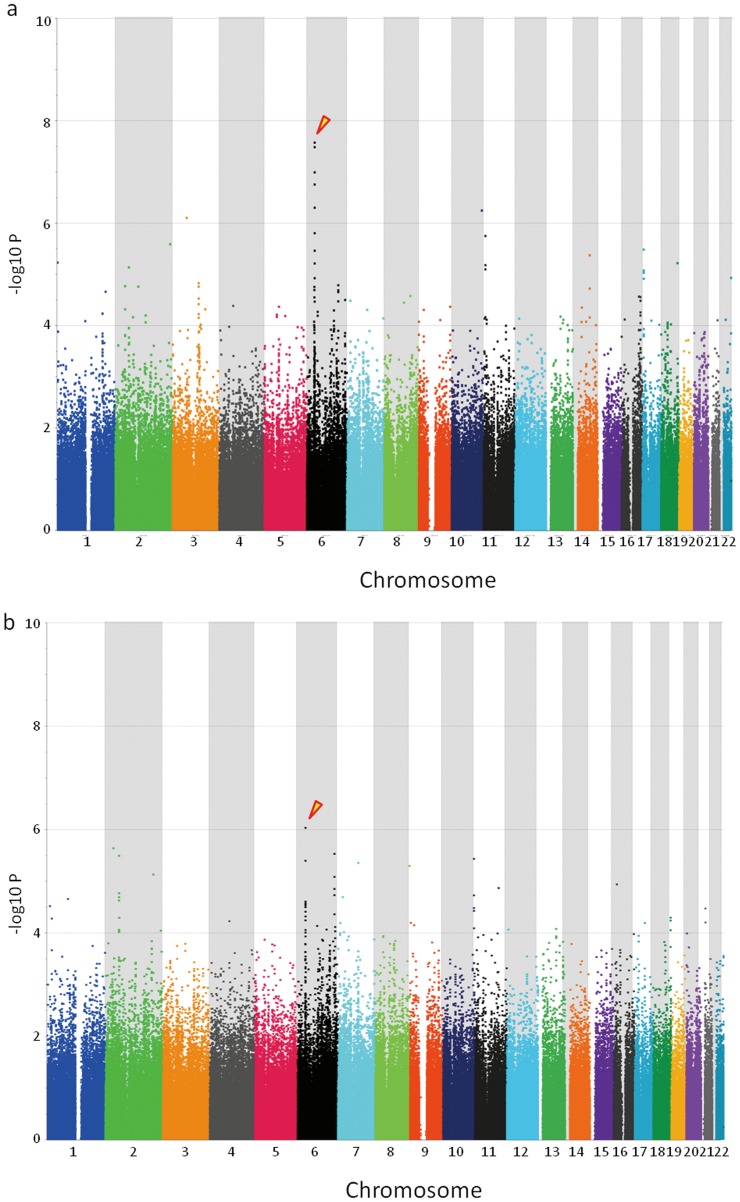
Results of genome-wide association studies. a) HBV carriers and healthy controls, and b) HBV carriers and HBV-resolved individuals were compared. *P* values were calculated by chi-squared test for allele frequencies. Dots with arrows on chromosome 6 show strong associations with protective effects against persistent HB infection and with HBV clearance.

In this study, we conducted a GWAS using samples from 181 Japanese HBV carriers (including asymptomatic carriers (ASC), CHB cases, LC cases and HCC cases, based on the criteria described in Materials and Methods) and 184 healthy controls in order to identify the host genetic factors related to progression of CHB. All samples were genotyped using a genome-wide SNP typing array (Affymetrix Genome-Wide Human SNP Array 6.0 for 900 K SNPs). Figure 1a shows a genome-wide view of the single point association data based on allele frequencies using the SNPs that met the following filtering criteria: (i) SNP call rate ≥95%; (ii) minor allele frequency (MAF) ≥1% for HBV carriers and healthy controls; and (iii) no deviation from Hardy-Weinberg equilibrium (HWE) *P*≥0.001 in healthy controls. We identified significant associations of protective effects against CHB with two SNPs (rs3077 and rs9277542) using the allele frequency model, both of which are located in the 3′ UTR of *HLA-DPA1* and in the sixth exon of *HLA-DPB1*, respectively (rs3077, *P* = 1.14×10^−7^, and rs9277542, *P* = 5.32×10^−8^, respectively). The association for rs9277542 reached a genome-wide level of significance in the GWAS panel (Bonferroni criterion *P*<8.36×10^−8^ (0.05/597,789)).

In order to validate the results of GWAS, a total of 32 SNPs, including the associated two SNPs (rs3077 and rs9277542), were selected for replication in two independent sets of HBV carriers and healthy controls (replication-1∶256 Japanese HBV carriers and 236 Japanese healthy controls; and replication-2∶344 Korean HBV carriers and 151 Korean healthy controls; Table 1). The associations for the original significant SNP (rs9277542) and marginal SNP (rs3077) on GWAS were replicated in both replication sets [replication-1 (Japanese); rs3077, *P* = 2.70×10^−8^, OR  = 0.48 and rs9277542, *P* = 3.33×10^−6^, OR  = 0.54; replication-2 (Korean); rs3077, *P* = 2.08×10^−6^, OR  = 0.47 and rs9277542, *P* = 8.29×10^−5^, OR  = 0.54, Table 2]. We conducted meta-analysis to combine these studies using the DerSimonian Laird method (random effects model) to incorporate variation among studies. As shown in Table 2, the odds ratios were quite similar across the three studies (GWAS and two replication studies) and no heterogeneity was observed (*P_het_* = 0.80 for rs3077 and 0.40 for rs9277542). *P_meta_* values were 4.40×10^−19^ for rs3077 (OR  = 0.46, 95% confidence interval (CI) = 0.39–0.54), and 1.28×10^−15^ for rs9277542 (OR  = 0.50, 95% CI = 0.43–0.60). Among the remaining 30 SNPs in the replication study, 27 SNPs were successfully genotyped by the DigiTag2 assay with SNP call rate ≥ 95% and HWE *p*-value ≥ 0.01. Two SNPs (rs9276431 and rs7768538), located in the genetic region including the *HLA-DQ* gene, were marginally replicated in the two sets of HBV carriers and healthy controls with Mantel-Haenszel *P* values of 2.80×10^−7^ (OR  = 0.56, 95% CI = 0.45–0.70) and 1.09×10^−7^ (OR  = 0.53, 95% CI = 0.42–0.67), respectively, when using additive, two-tailed Cochran Mantel-Haenszel (CMH) fixed-effects model with no evidence of heterogeneity (*P_het_* = 0.67 for rs9276431 and 0.70 for rs7768538) (Table S1).

**Table 1:** Number of study samples.

		GWAS	Replication-1	Replication-2
population		Japanese	Japanese	Korean
HBV carriers	Total	**181**	**256**	**344**
	IC	20	94	–
	CH	67	101	177
	LC	3	10	–
	HCC	91	51	167
Healthy controls		**184**	**236**	**151**
Resolved individuals		**185**	**150**	**106**

Abbreviation: IC, Inactive Carrier; CH, Chronic Hepatitis; LC, Liver Cirrhosis; HCC, Hepatocellular Carcinoma.

**Table 2:** Results of replication study for protective effects against CHB.

		Position		MAF^a^	Allele	Stage	HBV carriers			Healthy controls				OR^b^		
dbSNP rsID	Chr	Buld 36.3	Nearest Gene	(allele)	(1/2)	(population)	11	12	22	11	12	22	HWEp	95% CI	*P*-value^c^	*P_het_* d
rs3077	6	33141000	HLA-DPA1	0.44	T/C	GWAS	13	51	117	28	88	67	0.919	0.42	1.14×10^−7^	
				(T)		(Japanese)	(7.2)	(28.2)	(64.6)	(15.3)	(48.1)	(36.6)		(0.30–0.58)		
						Replication-1	26	95	134	46	125	65	0.309	0.48	2.70×10^−8^	
						(Japanese)	(10.2)	(37.3)	(52.5)	(19.5)	(53.0)	(27.5)		(0.37–0.62)		
						Replication-2	23	81	111	31	74	40	0.767	0.47	2.08×10^−6^	
						(Korean)	(10.7)	(37.7)	(51.6)	(21.4)	(51.0)	(27.6)		(0.35–0.65)		
						Meta-analysis^e^								0.46	4.40×10^−19^	0.80
														(0.39–0.54)		
rs9277542	6	33163225	HLA-DPB1	0.45	T/C	GWAS	18	53	110	29	102	52	0.073	0.42	5.32×10^−8^	
				(T)		(Japanese)	(9.9)	(29.3)	(60.8)	(15.8)	(55.7)	(28.4)		(0.31–0.58)		
						Replication-1	30	106	118	54	114	67	0.681	0.54	3.33×10^−6^	
						(Japanese)	(11.8)	(41.7)	(46.5)	(23.0)	(48.5)	(28.5)		(0.42–0.70)		
						Replication-2	30	87	94	35	72	36	0.933	0.54	8.29×10^−5^	
						(Korean)	(14.2)	(41.2)	(44.5)	(24.5)	(50.3)	(25.2)		(0.40–0.74)		
						Meta-analysis^e^								0.50	1.28×10^−15^	0.40
														(0.43–0.60)		

a Minor allele frequency and minor allele in 198 healthy Japanese (ref#19).

b Odds ratio of minor allele from two-by-two allele frequency table.

c P value of Pearson’s chi-square test for allelic model.

d Heterogeneity was tested using general variance-based method.

e Meta-analysis was tested using the random effects model.

Meta-analysis using the random effects model across 6 independent studies, including 5 additional published data, showed *P_meta_* = 3.94×10^−45^, OR  = 0.55 for rs3077, *P_meta_* = 1.74×10^−21^, OR  = 0.61 for rs9277535 and *P_meta_* = 1.69×10^−15^, OR  = 0.51 for rs9277542, with the SNP rs9277535 being located about 4-kb upstream from rs9277542 and showing strong linkage disequilibrium of r^2^ = 0.955 on the HapMap JPT (Table S2). As shown in Table S2, the odds ratio was very similar among the 6 studies, and heterogeneity was negligible with *P_het_* >0.01.

Moreover, based on GWAS using samples from 94 chronic HBV carriers with LC or HCC and 87 chronic HBV carriers without LC and HCC, we found no significant SNPs associated with CHB progression (Figure S1).

### Clearance of Hepatitis B virus in Japanese and Korean Individuals

We also conducted a GWAS to identify the host genetic factors related to clearance of HBV in the above 181 Japanese HBV carriers and 185 Japanese HBV-resolved individuals using a genome-wide SNP typing array (Affymetrix Genome-Wide Human SNP Array 6.0 for 900 K SNPs). The same two SNPs (rs3077 and rs9277542) showed strong associations in the allele frequency model (*P* = 9.24×10^−7^ and *P* = 3.15×10^−5^) with clearance of HBV (Figure 1b).

The above 32 SNPs, including the two associated SNPs (rs3077 and rs9277542), were selected for a replication study in two independent sets of HBV carriers and HBV resolved individuals (replication-1∶256 Japanese HBV carriers and 150 Japanese HBV resolved individuals; and replication-2∶344 Korean HBV carriers and 106 Korean HBV resolved individuals; Table 1). All 32 SNPs were genotyped using the DigiTag2 assay and 29 of 32 SNPs were successfully genotyped (Table S3). The associations of the original SNPs were replicated in both replication sets [replication-1 (Japanese): rs3077, *P* = 3.32×10^−2^, OR  = 0.72 and rs9277542, *P* = 1.25×10^−2^, OR  = 0.68; replication-2 (Korean): rs3077, *P* = 2.35×10^−7^, OR  = 0.41 and rs9277542, *P* = 4.97×10^−6^, OR  = 0.46; Table 3]. Meta-analysis using random effects model showed *P_meta_* = 1.56×10^−4^ for rs3077 (OR  = 0.51, 95% CI = 0.36–0.72), and 5.91×10^−7^ for rs9277542 (OR  = 0.55, 95% CI = 0.43–0.69). While there was evidence of heterogeneity between these studies for rs3077 (*P*_het_ = 0.03) and no evidence for rs9277542 (*P*_het_ = 0.19), significant associations with HBV clearance were observed with Mantel-Haenszel *P_meta_* = 3.28×10^−12^ for rs3077 and 1.42×10^−10^ for rs9277542, when using CMH fixed-effects model. Among the remaining 27 SNPs in the replication study, two SNPs (rs9276431 and rs7768538), located in a genetic region including *HLA-DQ* gene, were marginally replicated in the two sets of HBV carriers and HBV resolved individuals with Mantel-Haenszel *P* values of 2.10×10^−5^ (OR  = 0.59) and 1.10×10^−5^ (OR  = 0.56), respectively (Table S3), when using CMH fixed-effect model. Due to the existing heterogeneity among three groups (GWAS, Replication-1 and Replication-2) (*P_het_* = 0.03 for rs9276431 and 0.04 for rs7768538), weak associations were observed with *P_meta_* = 0.03 for rs9276431 and 0.02 for rs7768538 by the random effects model meta-analysis.

**Table 3:** Results of replication study for clearance of hepatitis B virus.

		Position		MAF^a^	Allele	Stage	HBV carriers			Resolved individuals			OR^b^		
dbSNP rsID	Chr	Buld 36.3	Nearest Gene	(allele)	(1/2)	(population)	11	12	22	11	12	22	95% CI	*P*-value^c^	*P_het_* d
rs3077	6	33141000	HLA-DPA1	0.44	T/C	GWAS	13	51	117	29	82	74	0.44	9.24×10^−7^	
				(T)		(Japanese)	(7.2)	(28.2)	(64.6)	(15.7)	(44.3)	(40.0)	(0.32–0.61)		
						Replication-1	26	95	134	20	64	60	0.72	3.32×10^−2^	
						(Japanese)	(10.2)	(37.3)	(52.5)	(13.9)	(44.4)	(41.7)	(0.53–0.97)		
						Replication-2	23	81	111	29	48	28	0.41	2.35×10^−7^	
						(Korean)	(10.7)	(37.7)	(51.6)	(27.6)	(45.7)	(26.7)	(0.29–0.58)		
						Meta-analysis^e^							0.51	1.56×10^−4^	0.03
													(0.36–0.72)		
						Meta-analysis^e^							0.43	1.89×10^−12^	0.75
						(GWAS+replication-2)							(0.34–0.54)		
rs9277542	6	33163225	HLA-DPB1	0.45	T/C	GWAS	18	53	110	28	88	69	0.51	3.15×10^−5^	
				(T)		(Japanese)	(9.9)	(29.3)	(60.8)	(15.1)	(47.6)	(37.3)	(0.37–0.70)		
						Replication-1	30	106	118	28	62	52	0.68	1.25×10^−2^	
						(Japanese)	(11.8)	(41.7)	(46.5)	(19.7)	(43.7)	(36.6)	(0.51–0.92)		
						Replication-2	30	87	94	30	53	22	0.46	4.97×10^−6^	
						(Korean)	(14.2)	(41.2)	(44.5)	(28.6)	(50.5)	(21.0)	(0.33–0.64)		
						Meta-analysis^e^							0.55	5.91×10^−7^	0.19
													(0.43–0.69)		
						Meta-analysis^e^							0.49	9.69×10^−10^	0.65
						(GWAS+replication-2)							(0.39–0.61)		

a Minor allele frequency and minor allele in 198 healthy Japanese (ref#19).

b Odds ratio of minor allele from two-by-two allele frequency table.

c P value of Pearson’s chi-square test for allelic model.

d Heterogeneity was tested using general variance-based method.

e Meta-analysis was tested using the random effects model.

Meta-analysis across 6 independent studies, including 5 additional published data, showed *P_meta_* = 1.48×10^−9^, OR  = 0.60 for rs3077, *P_meta_* = 1.08×10^−17^, OR  = 0.66 for rs9277535 and *P_meta_* = 5.14×10^−5^, OR  = 0.55 for rs9277542 (Table S4). As shown in Table S4, the OR for the rs9277535 and rs9277542 were similar among the 6 independent studies, and heterogeneity was negligible (*P_het_* = 0.03 for rs9277535 and 0.14 for rs9277542). However, significant level of heterogeneity for rs3077 was observed with *P_het_* = 9.57×10^−6^ across 5 independent studies, including our study.

### URLs

The results of the present GWAS are registered at a public database: https://gwas.lifesciencedb.jp/cgi-bin/gwasdb/gwas_top.cgi.

## Discussion

The recent genome-wide association study showed that the SNPs located in a genetic region including *HLA-DPA1* and *HLA-DPB1* genes were associated with chronic HBV infection in the Japanese and Thai population [10], [11]. In this study, we confirmed a significant association between SNPs (rs3077 and rs9277542) located in the same genetic region as *HLA-DPA1* and *HLA-DPB1* and protective effects against CHB in Korean and Japanese individuals. Mata-analysis using the random effects model across 6 independent studies including our study suggested that, widely in East Asian populations, variants in antigen binding sites of *HLA-DP* contribute to protective effects against persistent HBV infection (Table S2).

On GWAS and replication analysis with Japanese and Korean individuals, we identified associations between the same SNPs (rs3077 and rs9277542) in the *HLA-DPA1* and *HLA-DPB1* genes and HBV clearance; however, no new candidate SNPs from the GWAS were detected on replication analysis (Table S3). When the data of reference#18 was excluded from the meta-analysis across 6 independent studies, heterogeneity among 4 studies was estimated to be *P_het_* = 0.15 and significant association of rs3077 with HBV clearance was observed with *P_meta_* = 5.88×10^−24^, OR  = 0.56 (Table S4). In our study, a negligible level of heterogeneity for rs3077 was also observed (*P_het_* = 0.03) on meta-analysis by adding replication-1 (Table 3). Despite the heterogeneity in replication-1, a marginal association was observed for rs3077 with the same downward trend in the odds ratio (*P* = 3.32×10^−2^, OR  = 0.72). Moreover, meta-analysis using GWAS and replication-2 showed significant association of *P_meta_* = 1.89×10^−12^, OR  = 0.43 for rs3077 with no evidence of heterogeneity (*P_het_* = 0.75). Although the reason why heterogeneity was observed in replication-1 is unclear, one possible reason is the clinical heterogeneity due to different kits being used for antibody testing. The associations of *HLA-DPA1*/−*DPB1* with CHB and HBV clearance showed the same level of significance in the comparison of HBV patients with HBV resolved individuals (OR  = 0.43 for rs3077 and 0.49 for rs9277542) as the one with healthy controls (OR  = 0.46 for rs3077 and 0.50 for rs9277542), when the replication-1 was excluded in the analysis (Table 2 and Table 3). The results of meta-analysis across 6 independent studies including our study also showed the same or slightly weaker associations in the comparison of HBV patients with HBV resolved individuals (OR  = 0.56 for rs3077, 0.66 for rs9277535 and 0.55 for rs9277542) than in the one with healthy controls (OR  = 0.55 for rs3077, 0.61 for rs9277535 and 0.51 for rs9277542), which was the opposite result as we expected (Table S2 and Table S4). These results may suggest that other unknown immune system(s) exist to eliminate the HBV in the HBV resolved individuals.

Among the HLA class II loci (*HLA-DPA1*, *HLA-DPB1* and *HLA-DQB2*), which were associated with CHB and HBV clearance, a weak linkage disequilibrium (r^2^<0.1) was observed between *HLA-DQB2* locus and *HLA-DPA1*/−*DPB1* loci in Japanese and Korean populations (Figure S2). We also found that similar linkage disequilibrium blocks (r^2^) were observed among three subgroups (HBV carriers, HBV resolved individuals and Healthy controls). Moreover, logistic regression analysis of *HLA-DP* (rs3077 and rs92775542) with use of *HLA-DQ* (rs9276431 and rs768538) as covariates showed that the same level of significant associations of *HLA-DP* with CHB and HBV clearance as shown in the single-point association analysis, while no associations of *HLA-DQ* with *P_log_* >0.05 were detected both in Japanese and in Korean (Table S5). These results show that *HLA-DP* is the main genetic factor for susceptibility to CHB and HBV clearance, and the associations of *HLA-DQB2* would result from linkage disequilibrium of *HLA-DP*A1/−DPB1.

In this study, we confirmed the significant associations between *HLA-DPA1* and *HLA-DPB1*, and protective effects against CHB and HBV clearance in Japanese and Korean individuals. These results suggest that the associations between the *HLA-DP* locus, CHB and HBV clearance are widely replicated in East Asian populations, including Chinese, Thai, Japanese and Korean individuals; however, there have been no similar GWAS performed in Caucasian and African populations. Moreover, there were no significant SNPs associated with HCC development in this study, thus suggesting that it is necessary to increase the sample size. To clarify the pathogenesis of CHB or the mechanisms of HBV clearance, further studies are necessary, including a functional study of the *HLA-DP* molecule, identification of novel host genetic factors other than *HLA-DP*, and variation analysis of HBV.

## Materials and Methods

### Ethics Statement

All study protocols conform to the relevant ethical guidelines, as reflected in the *a priori* approval by the ethics committees of all participating universities and hospitals. The written informed consent was obtained from each patient who participated in this study and all samples were anonymized.

### Genomic DNA Samples and Clinical Data

All of the 1,793 Japanese and Korean samples, including individuals with CHB, healthy controls and HBV-resolved individuals (HBsAg-negative and anti-HBc-positive), were collected at 20 multi-center hospitals (liver units with hepatologists) throughout Japan and Korea. The 19 hospitals in Japan were grouped into the following 8 areas: Hokkaido area (Hokkaido University Hospital, Teine Keijinkai Hospital), Tohoku area (Iwate Medical University Hospital), Kanto area (Musashino Red Cross Hospital, Saitama Medical University, Kitasato University Hospital, University of Tokyo), Koshin area (Shinshu University Hospital, Kanazawa University Hospital), Tokai area (Nagoya City University Hospital, Nagoya Daini Red Cross Hospital), Kinki area (Kyoto Prefectural University of Medicine Hospital, National Hospital Organization Osaka National Hospital, Osaka City University), Chugoku/Shikoku area (Tottori University Hospital, Ehime University Hospital, Yamaguchi University Hospital, Kawasaki Medical College Hospital) and Kyushu area (Kurume University Hospital). Korean samples were collected at Yonsei University College of Medicine.

HBV status was measured based on serological results for HBsAg and anti-HBc with a fully automated chemiluminescent enzyme immunoassay system (Abbott ARCHITECT; Abbott Japan, Tokyo, Japan, or LUMIPULSE f or G1200; Fujirebio, Inc., Tokyo, Japan). For clinical staging, inactive carrier (IC) state was defined by the presence of HBsAg with normal ALT levels over 1 year (examined at least four times at 3-month intervals) and without evidence of portal hypertension. Chronic hepatitis (CH) was defined by elevated ALT levels (>1.5 times the upper limit of normal [35 IU/L]) persisting over 6 months (at least by 3 bimonthly tests). Liver cirrhosis (LC) was diagnosed principally by ultrasonography (coarse liver architecture, nodular liver surface, blunt liver edges and hypersplenism), platelet counts <100,000/cm^3^, or a combination thereof. Histological confirmation by fine-needle biopsy of the liver was performed as required. Hepatocellular carcinoma (HCC) was diagnosed by ultrasonography, computerized tomography, magnetic resonance imaging, angiography, tumor biopsy or a combination thereof.

The Japanese control samples from HBV-resolved subjects (HBsAg-negative and anti-HBc-positive) at Nagoya City University-affiliated healthcare center were used by comprehensive agreement (anonymization in an unlinkable manner) in this study. Some of the unrelated Japanese healthy controls were obtained from the Japan Health Science Research Resources Bank (Osaka, Japan). One microgram of purified genomic DNA was dissolved in 100 µl of TE buffer (pH 8.0) (Wako, Osaka, Japan), followed by storage at −20°C until use.

### SNP Genotyping and Data Cleaning

For GWAS, we genotyped a total of 550 individuals, including 181 Japanese HBV carriers, 184 Japanese healthy controls and 185 spontaneously HBV-resolved Japanese individuals (HBsAg-negative and anti-HBc-positive), using the Affymetrix Genome-Wide Human SNP Array 6.0 (Affymetrix, Inc., Santa Clara, CA), in accordance with the manufacturer’s instructions. The average QC call rate for 550 samples reached 98.47% (95.00–99.92%), which had an average sample call rate of 98.91% (93.55–99.74%) by determining the genotype calls of over 900 K SNPs using the Genotyping Console v4.1 software (with Birdseed v1 algorithm) provided by the manufacturer [19]. We then applied the following thresholds for SNP quality control in data cleaning: SNP call rate ≥95% and MAF ≥1% for three groups (HBV carriers, healthy controls and HBV-resolved individuals), and HWE *P*-value ≥0.001 for healthy controls [20]. Here, SNP call rate is defined for each SNP as the number of successfully genotyped samples divided by the number of total samples genotyped. A total of 597,789 SNPs and 590,278 SNPs on autosomal chromosomes passed the quality control filters in the genome-wide association analysis using HBV carriers and healthy controls, and using HBV carriers and HBV-resolved individuals, respectively (Figure 1). All cluster plots for the SNPs showing *P*<0.0001 on association analyses in the allele frequency model were confirmed by visual inspection, and SNPs with ambiguous cluster plots were excluded.

In the following replication stage, we selected a set of 32 SNPs with *P*<0.0001 in the GWAS using HBV carriers and HBV-resolved individuals. SNP genotyping in two independent sets of 256 Japanese HBV carriers, 236 Japanese healthy controls and 150 Japanese HBV-resolved individuals (Table 1, replication-1), and 344 Korean HBV carriers, 151 Korean healthy controls and 106 Korean HBV-resolved individuals (Table 1, replication-2) was completed for the selected 32 SNPs using the DigiTag2 assay [21], [22] and custom TaqMan SNP Genotyping Assays (Applied Biosystems, Foster City, CA) on the LightCycler 480 Real-Time PCR System (Roche, Mannheim, Germany).

### Statistical Analysis

The observed associations between SNPs and the protective effects on chronic hepatitis B or clearance of hepatitis virus B were assessed by chi-squared test with a two-by-two contingency table in allele frequency model. SNPs on chromosome X were removed because gender was not matched among HBV carriers, healthy controls and HBV-resolved individuals. A total of 597,789 SNPs and 590,278 SNPs passed the quality control filters in the GWAS stage; therefore, significance levels after Bonferroni correction for multiple testing were *P* = 8.36×10^−8^ (0.05/597,789) and *P* = 8.47×10^−8^ (0.05/590,278), respectively. For the replication study, 29 of 32 SNPs were successfully genotyped; therefore, we applied *P* = 0.0017 (0.05/29) as a significance level, and none of the 29 markers genotyped in the replication stage showed deviations from the Hardy-Weinberg equilibrium in healthy controls (*P*>0.01).

The genetic inflation factor λ was estimated by applying the Cochrane-Armitage test on all SNPs and was found to be 1.056 and 1.030 in the GWAS using HBV carriers and healthy controls, and using HBV carriers and HBV-resolved individuals, respectively (Figure S3). These results suggest that the population substructure should not have any substantial effect on statistical analysis. In addition, the principal component analysis in a total of 550 individuals in the GWAS stage together with the HapMap samples also revealed that the effect of population stratification was negligible (Figure S4).

Based on the genotype data of a total of 1,793 samples including 1,192 Japanese samples and 601 Korean samples in both GWAS and replication stages, haplotype blocks were estimated using the Gabriel’s algorithm using the Haploview software (v4.2) (Figure S2). In the logistic regression analysis, two SNPs (rs9276431 and rs7768538) within the HLA-DQ locus were individually involved as a covariate (Table S5). Statistical analyses were performed using the SNP & Variation Suite 7 software (Golden Helix, MT, USA).

## Supporting Information

Figure S1 **GWAS using samples from HBV carriers with LC or HCC, and HBV carriers without LC and HCC.** *P* values were calculated using chi-squared test for allele frequencies.
(PPTX)

Figure S2 **Estimation of linkage disequilibrium blocks in HBV patients, HBV resolved individuals and healthy controls in Japanese and Korean.** The LD blocks (r^2^) were analyzed using the Gabriel’s algorithm.
(PPTX)

Figure S3 **Quantile-quantile plot for test statistics (allele-based chi-squared tests) for GWAS results.** Dots represent P values of each SNP that passed the quality control filters. Inflation factor λ was estimated to be: a) 1.056 in the analysis with HBV carriers and healthy controls; and b) 1.030 with HBV carriers and HBV-resolved individuals.
(PPTX)

Figure S4 **Principal component analysis on a total of 550 individuals in GWAS, together with HapMap samples (CEU, YRI and JPT).**
(PPTX)

Table S1 **Results for 29 SNPs selected in replication study using samples of HBV carriers and healthy controls.** ^a^ *P* values by chi-squared test for allelic model. ^b^Odds ratio of minor allele from two-by-two allele frequency table. ^c^Meta-analysis was tested using additive, two-tailed CMH fixed-effects model.
(XLSX)

Table S2 **Results of meta-analysis for protective effects against persistent HB infection across 6 independent studies, including this study.** ^a^Minor allele frequency and minor allele in 198 healthy Japanese (ref#19). ^b^Odds ratio of minor allele from two-by-two allele frequency table. ^c^ *P* value of Pearson’s chi-squared test for allele model. ^d^Heterogeneity was tested using general variance-based method. ^e^Meta-analysis was tested using the random effects model.
(XLSX)

Table S3 **Results for 29 SNPs selected in replication study using samples from HBV carriers and HBV-resolved individuals.** ^a^ *P* values by chi-squared test for allelic model. ^b^Odds ratio of minor allele from two-by-two allele frequency table. ^c^Meta-analysis was tested using additive, two-tailed CMH fixed-effects model.
(XLSX)

Table S4 **Results of meta-analysis for clearance of HBV across 6 independent studies, including this study.** ^a^Minor allele frequency and minor allele in 198 healthy Japanese (ref#19). ^b^Odds ratio of minor allele from two-by-two allele frequency table. ^c^ *P* value of Pearson’s chi-squared test for allele model. ^d^Heterogeneity was tested using general variance-based method. ^e^Meta-analysis was tested using the random effects model.
(XLSX)

Table S5 **Logistic regression analysis of** ***HLA-DP*** **(rs3077 and rs9277542) and** ***HLA-DQ*** **(rs9276431 and rs7768538) with susceptibility to CHB and HBV clearance using the** ***HLA-DQ*** **genotypes individually as a covariate.**
(XLSX)
