# Supplementary figures and images for: Genome-Wide Association Study Confirming Association of HLA-DP with Protection against Chronic Hepatitis B and Viral Clearance in Japanese and Korean
Source: PLoS One. 2012 Jun 21;7(6):e39175. doi: 10.1371/journal.pone.0039175 (PMC3380898; doi:10.1371/journal.pone.0039175)

## Slide 1
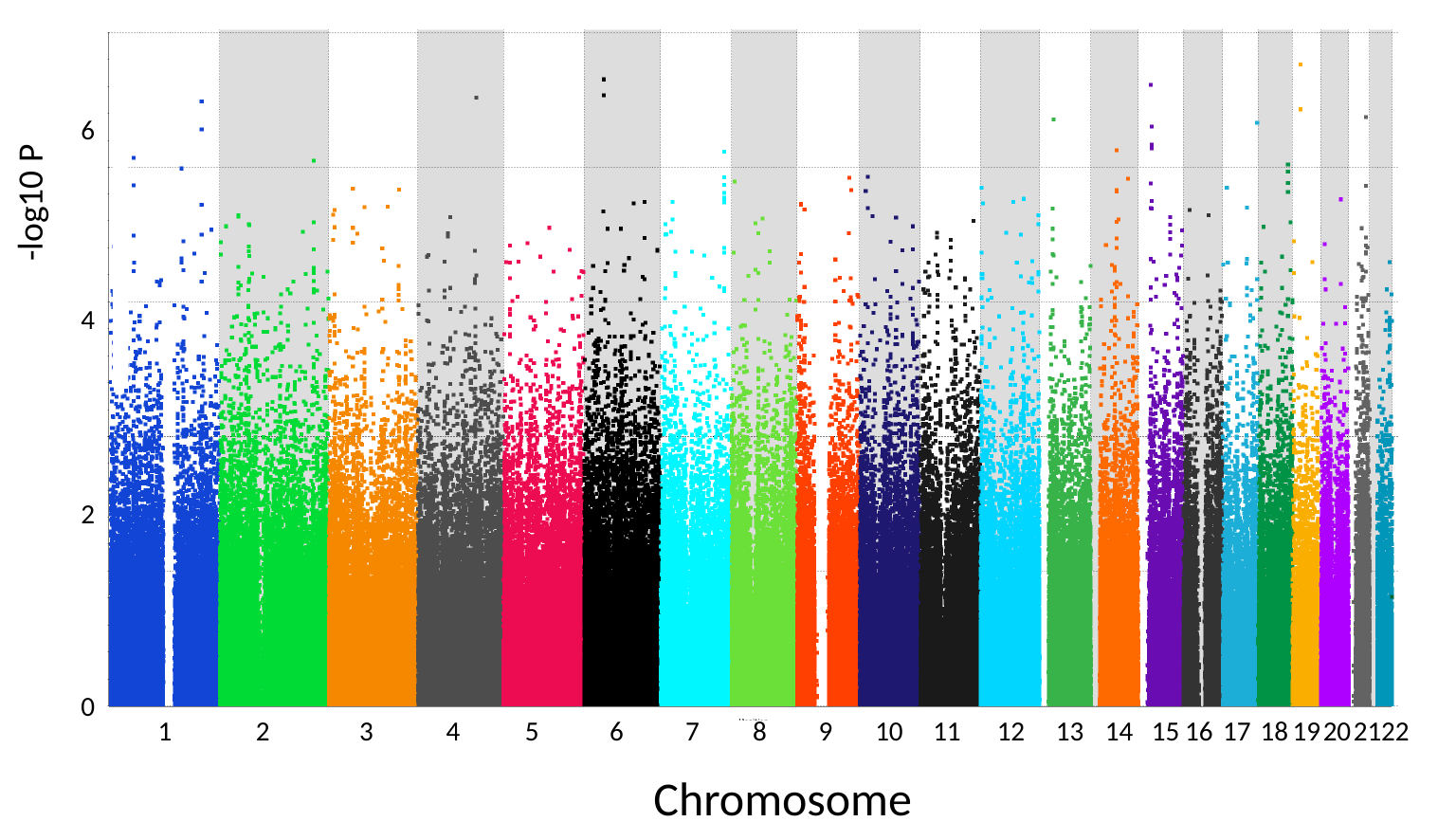

6
-log10 P
4
2
0
1
2
3
4
5
6
7
8
9
10
11
12
13
14
15
16
17
18
19
20
21
22
Chromosome

Supplement: Figure S1 — GWAS using samples from HBV carriers with LC or HCC, and HBV carriers without LC and HCC. P values were calculated using chi-squared test for allele frequencies. (PPTX) [file pone.0039175.s001.pptx]

## Slide 1
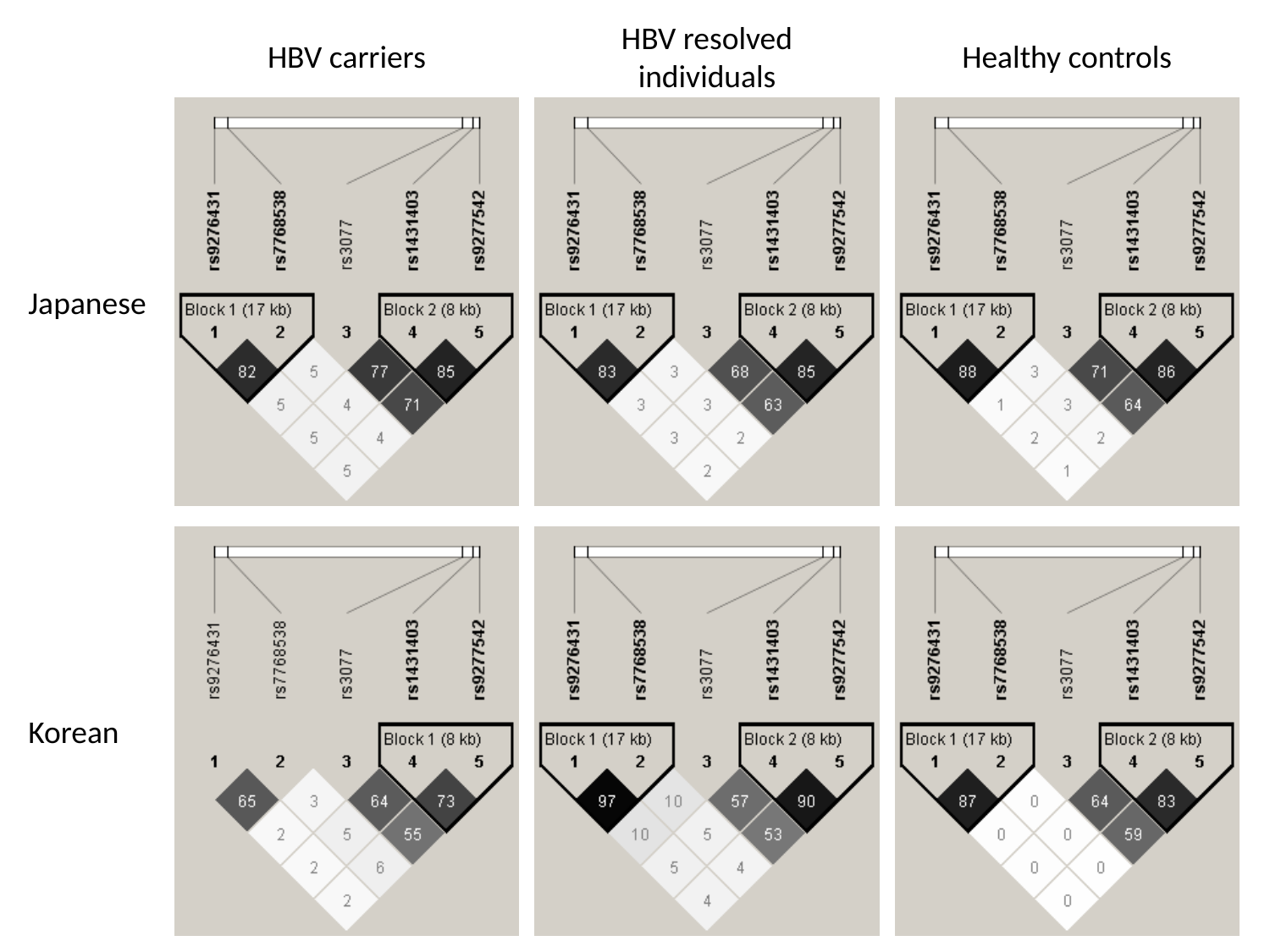

HBV resolved
individuals
HBV carriers
Healthy controls
Japanese
Korean

Supplement: Figure S2 — Estimation of linkage disequilibrium blocks in HBV patients, HBV resolved individuals and healthy controls in Japanese and Korean. The LD blocks (r2) were analyzed using the Gabriel’s algorithm. (PPTX) [file pone.0039175.s002.pptx]

## Slide 1
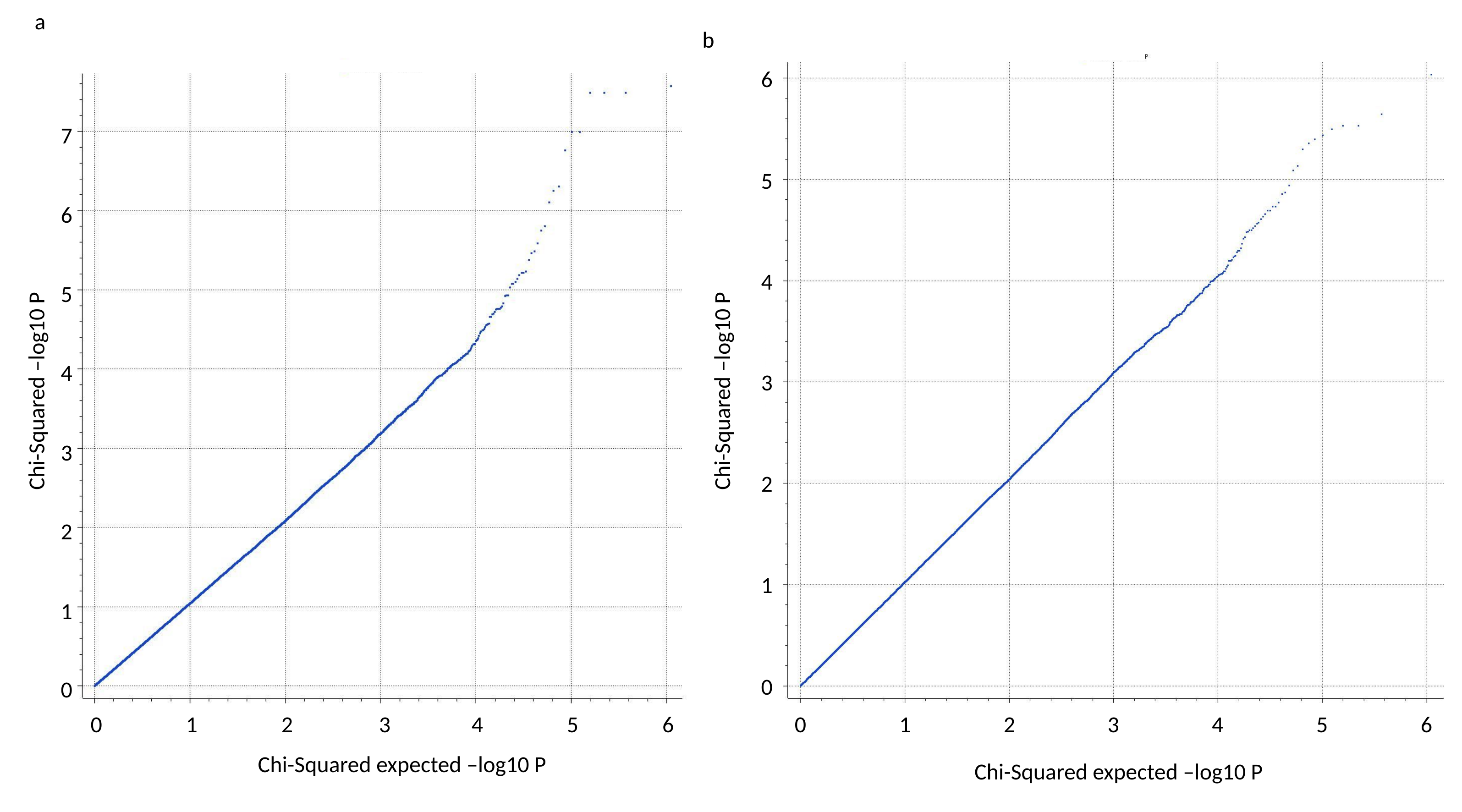

a
b
6
7
5
6
4
5
4
3
Chi-Squared –log10 P
Chi-Squared –log10 P
3
2
2
1
1
0
0
0
1
2
3
4
5
6
0
1
2
3
4
5
6
Chi-Squared expected –log10 P
Chi-Squared expected –log10 P

Supplement: Figure S3 — Quantile-quantile plot for test statistics (allele-based chi-squared tests) for GWAS results. Dots represent P values of each SNP that passed the quality control filters. Inflation factor λ was estimated to be: a) 1.056 in the analysis with HBV carriers and healthy controls; and b) 1.030 with HBV carriers and HBV-resolved individuals. (PPTX) [file pone.0039175.s003.pptx]

## Slide 1
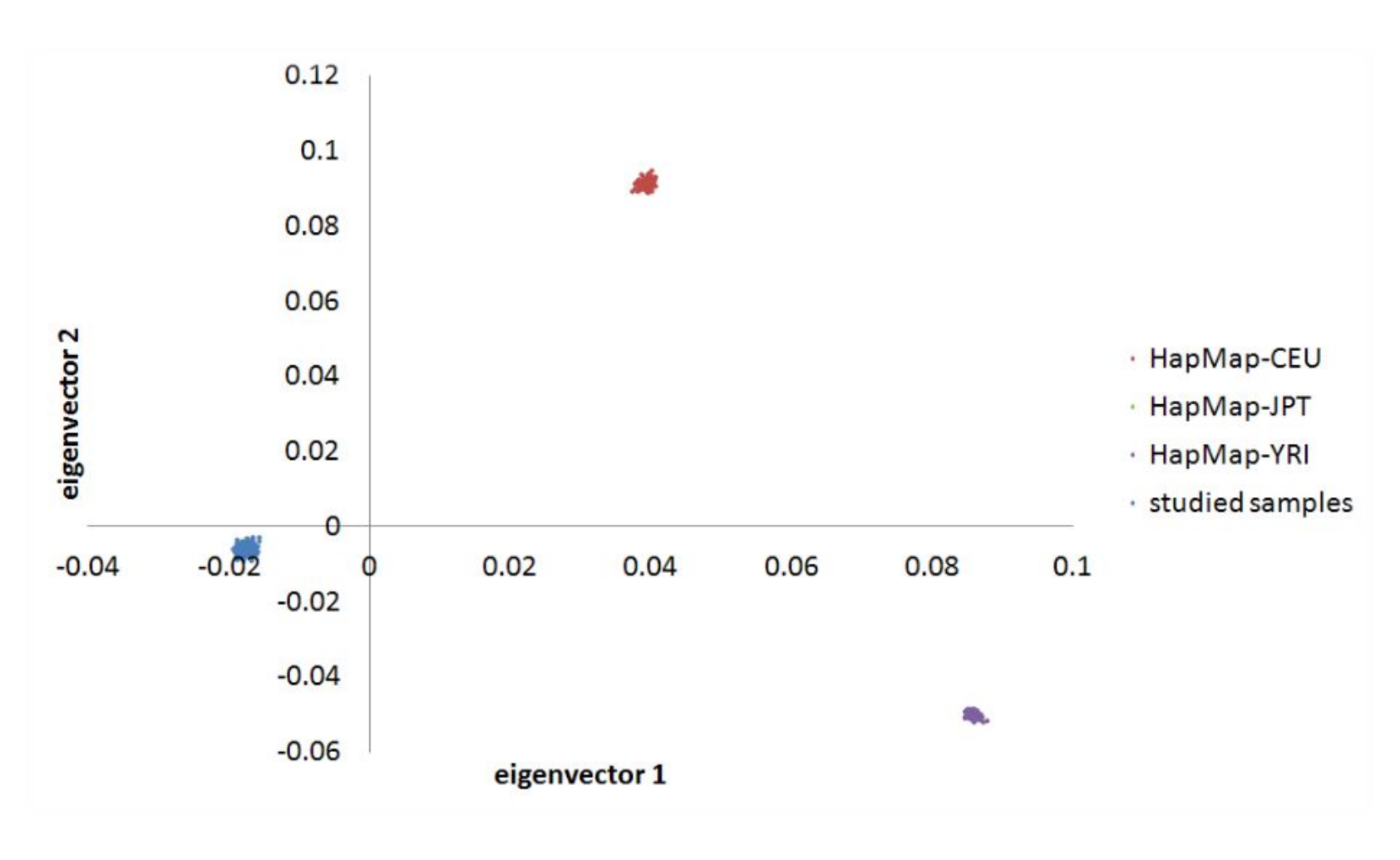

Supplement: Figure S4 — Principal component analysis on a total of 550 individuals in GWAS, together with HapMap samples (CEU, YRI and JPT). (PPTX) [file pone.0039175.s004.pptx]
